# Reforestation of *Cunninghamia lanceolata* changes the relative abundances of important prokaryotic families in soil

**DOI:** 10.3389/fmicb.2024.1312286

**Published:** 2024-02-13

**Authors:** Xue-Yan Hou, Wen-Tao Qiao, Ji-Dong Gu, Chao-Ying Liu, Muhammad Mahroz Hussain, Dao-Lin Du, Yi Zhou, Yong-Feng Wang, Qian Li

**Affiliations:** ^1^Institute of Environmental Health and Ecological Security, School of Environment and Safety Engineering, Jiangsu University, Zhenjiang, China; ^2^Environmental Engineering, Guangdong Technion Israel Institute of Technology, Shantou, China; ^3^Jingjiang College, School of Environment and Safety Engineering, Jiangsu University, Zhenjiang, China; ^4^Guangdong Provincial Key Laboratory of Silviculture, Protection and Utilization, Guangdong Academy of Forestry, Guangzhou, China

**Keywords:** prokaryotic community, land use change, reforestation, *Cunninghamia lanceolata*, soil organic matter, nitrogen

## Abstract

Over the past decades, many forests have been converted to monoculture plantations, which might affect the soil microbial communities that are responsible for governing the soil biogeochemical processes. Understanding how reforestation efforts alter soil prokaryotic microbial communities will therefore inform forest management. In this study, the prokaryotic communities were comparatively investigated in a secondary Chinese fir forest (original) and a reforested Chinese fir plantation (reforested from a secondary Chinese fir forest) in Southern China. The results showed that reforestation changed the structure of the prokaryotic community: the relative abundances of important prokaryotic families in soil. This might be caused by the altered soil pH and organic matter content after reforestation. Soil profile layer depth was an important factor as the upper layers had a higher diversity of prokaryotes than the lower ones (*p* < 0.05). The composition of the prokaryotic community presented a seasonality characteristic. In addition, the results showed that the dominant phylum was Acidobacteria (58.86%) with Koribacteraceae (15.38%) as the dominant family in the secondary Chinese fir forest and the reforested plantation. Furthermore, soil organic matter, total N, hydrolyzable N, and ${\text{NH}}_{4}^{+}-\text{N}$ were positively correlated with prokaryotic diversity (*p* < 0.05). Also, organic matter and ${\text{NO}}_{3}^{-}-\text{N}$ were positively correlated to prokaryotic abundance (*p* < 0.05). This study demonstrated that re-forest transformation altered soil properties, which lead to the changes in microbial composition. The changes in microbial community might in turn influence biogeochemical processes and the environmental variables. The study could contribute to forest management and policy-making.

## Highlights

- Acidobacteria dominated subtropical Chinese fir forest soil.
- Forest reformation altered the structure of the prokaryotic community.
- Soil layer influenced prokaryotic community composition.
- Prokaryotic community composition showed seasonality.

## 1 Introduction

Forests are one of the most important ecosystems carrying out many ecological and economic functions, such as CO_2_ sequestration, conserving natural diversity, protecting environment, and providing sustainable timber resources. Forest soils are the favorite habitat for most microorganisms, which are involved in a wide range of elemental biogeochemical cycles, such as nitrogen (N; Stein and Klotz, 2016), carbon (C), and sulfur (S) cycles (Kertesz and Mirleau, 2004). Microbial composition and activities are substantially affected by biotic and abiotic factors (Llado et al., 2018). Thus, it is necessary to explore the effects of these biotic and abiotic factors on soil microorganisms under the background that human activities are influencing forests globally.

Land use change could impact vegetation (Tasser and Tappeiner, 2002; Shen et al., 2023), and thus the soil micro- and macro-biota (De Graaff et al., 2014; Navarrete et al., 2015; Poirier et al., 2016; Li et al., 2022). Nowadays, many forests have been converted to croplands (Bruun et al., 2013) or commercial tree stands (Prabowo et al., 2017). In southern China some secondary forests have even been completely cut down and replaced by a single commercial tree species (Wang et al., 2010, 2011; Guan et al., 2015). Many of these forests have low quality because of poor management practices, for example, some forests possess low water holding capacity (Zeng et al., 2011) and pathogens attack is also among the major threats to the forest trees sustainability (Durán et al., 2008). There is a dire need to reform these forests by felling the diseased trees and replanting new seedlings in the forest gaps. A radical practice is clear cutting and then the cutover land is planted with single tree species. However, these kinds of intense land use changes will greatly disturb the forest soils and native microorganism communities (Zhang, 1995). The soil biogeochemical processes might be impacted due to soil microbial community disturbance which can influence the environmental cycling and natural ecosystems. To our knowledge, a limited number of studies have been conducted to address such an emerging issue and minimum work has been reported to overcome the microbial interaction and its role in forest biogeochemical cycling (Verchot et al., 1999, 2000).

We have carried out studies on some pivotal prokaryotic communities, including ammonia-oxidizing archaea and bacteria (Wu et al., 2018), anammox bacteria and n-damo bacteria (Gan et al., 2016; Meng et al., 2016), diazotrophs (Meng et al., 2018), denitrifying microorganisms (Meng et al., 2017), in the Nanling National Nature Reserve in Guangdong province, China. These articles documented the prokaryotic community in original forests, but the effects of forest transformation on the entire prokaryotic community have not been explored. In this study, we further investigated the entire prokaryotic community in a secondary Chinese fir forest (original) and a newly planted Chinese fir plantation from the cutover land of a secondary Chinese fir forest in the Nanling National Nature Reserve. We aimed to explore how this kind of forest transformation affects the prokaryotic community and the underlying mechanisms, providing important management recommendations for reforestation. We hypothesized that forest conversion change tree species, leading to different litter quality. Increased human activity could also disturb soils, which impact soil organic content and pH. The changes in soil properties then result in the variation in microbial community.

## 2 Materials and methods

### 2.1 Study sites and soil sample collection

Soils were collected from Guangdong Nanling National Nature Reserve (24°37′-24°57′N, 112°30′-113°04′E), whose climate is subtropical monsoon with an annual precipitation of 2,108.4 mm. Two types of forests were selected: one was a secondary Chinese fir forest (*Cunninghamia lanceolata*), which was 26 years old. Part of the above forest was clear cut and re-planted with Chinese fir seedlings 6 years ago. This new plantation was selected as the second site. Three replicate sampling points were randomly collected from each type of forest, respectively in winter (8 January 2015) and summer (5 August 2015). At each sampling point, the soil samples were separately collected from the upper (0–2 cm) and lower (18–20 cm) horizons, respectively representing A and B horizons. About 3 kg of soil was collected and homogenized for each sample. Approximately 900 g of homogeneous soil was used for physicochemical analysis and 100 g for subsequent molecular analysis. Samples were immediately placed in a cooling box with ice bags before laboratory physicochemical analysis. In the laboratory, samples for molecular studies were stored at −80°C, and samples for physicochemical analysis were processed immediately.

### 2.2 Assay of soil physicochemical properties

The physicochemical properties of the soil samples were analyzed by the Guangdong Institute of Ecology, Environment and Soil Science using the Methods of Agricultural Chemical Analysis (Rushen, 2000). Soil pH values were measured by a pH meter (Starter 3C, OHAUS) while organic carbon was determined using the dichromate sulfate ablation method, total nitrogen by Kjeldahl, active nitrogen by KOH diffusion, ammonia and nitrate nitrogen by potassium chloride extraction, and total phosphorus by digestion with HClO_4_ + H_2_SO_4_ followed by a colorimetric method. The effective phosphorus was first extracted by NaHCO_3_ and then the effective phosphorus content was determined by the colorimetric method. A UV-Vis spectrophotometer (model 752 N, Jinko, Shanghai) was used for all spectrophotometric determinations. 2 M KCl was used to extract exchangeable aluminum from the soil samples, and the concentrations of exchangeable aluminum were determined by ICP-OES (Perkin Elmer Optima 8300, Waltham, MA, USA). The results of soil parameters are given in Supplementary Table 1.

### 2.3 Total genomic DNA extraction and 16s rRNA gene sequencing

Using the SoilMaster DNA extraction kit to extract Genomic DNA in duplicate from each soil sample (Epicenter Biotechnologies, Madison, WI). Using a NanoDrop spectrophotometer to examine DNA concentration and quality. After extracting, DNA from the same sample was mixed and diluted to 10 ng μL^−1^ and stored at −40°C for downstream use.

The hypervariable region of 16S rRNA gene V4-V5 was amplified with 515F (5′-GTGCCAGCMGCCGCGGTAA-3′) and 909R (5′-CCCCGYCAATTCMTTTRAGT-3′) as general primers. The PCR reaction (25 μl) consisted of 5 ng DNA, 1 unit of EX Taq (TaKaRa, Dalian, China), 1 buffer of EX Taq, 0.2 mM for each dNTP, and 0.4 μM for each primer. Amplification conditions consisted of an initial denaturation step of 94°C for 5 min, followed by 30 cycles of 94°C for 30 s, 55°C for 30 s, 72°C for 50 s, and finally an extension of 10 min at 72°C. The PCR reaction was repeated for each sample, and the products were pooled and subjected to 1% agarose gel electrophoresis. Bands of the correct size were taken and purified using the SanPrep DNA Gel extraction kit (Sangon Biotech, Shanghai, China). All PCR products were quantified by Nanodrop and mixed with an equal molar amount from each sample. Sequencing samples were prepared using the TruSeq DNA kit according to the manufacturer's instructions. The purified library was diluted, denatured, and re-diluted according to Illumina library preparation protocol, mixed with PhiX (equal to 30% of the final DNA amount), and sequenced using Reagent Kit v2 2 × 250 bp Illumina Miseq system based on environmental genomics platform of Chengdu Institute of Biology.

### 2.4 High-throughput sequence data analysis

Using QIIME Pipeline-Version 1.7.0 process sequence data (http://qiime.org/). Trim the sequence reading and assign it to the sample according to its barcode. High-quality sequences with length >300 bp, unambiguous base N and average base quality score >30 were selected for downstream analysis. The sequences were clustered into operational classification units (OTUs) under the recognition threshold of 97%. The Uchime algorithm was used for chimeric check of the aligned 16S rRNA gene sequences (Edgar et al., 2011). A total of 9,190 reads were randomly retaken from all samples, and the α-diversity, phylogenetic distance of the whole tree, Chao1 estimates of richness, species, and Shannon diversity index were calculated. Generate sparse curves from observed species. The ribosome database item classifier was used for classification (Wang et al., 2007).

### 2.5 Principal coordinate analysis and redundancy analysis

Based on 6,408 OTUs that appeared at least three times in 24 samples, principal coordinate analysis (PCoA) was performed using Canoco 5.0 software to assess overall structural changes between samples (Brackin et al., 2013).

### 2.6 Statistical analysis

Using Microsoft Excel 2019 software, a two-factor analysis of variance (ANOVA) was used to analyze the effects of the soil layer, reforestation type, and season on soil properties, prokaryotic biodiversity, abundance and the 10 most abundant families of prokaryotes. The value *p* < 0.05 or 0.01 was considered statistically significant at two levels. The correlation coefficient (*r*) between soil physicochemical parameters, soil physicochemical parameters and the ten most abundant families were calculated using the following formula;


$$r=\frac{n\left(\sum XY\right)-(\sum X)(\sum Y)}{\sqrt{\left[n\sum X^{2}\right]-{\left(\sum X\right)}^{2}\left[\left[n\sum Y^{2}\right]-{\left(\sum Y\right)}^{2}\right]}}$$

X: every x-variable value, Y: every y-variable value, n: sample size.

The diversity significance difference (*p*-value) of Chao1, Observed OTU and Shannon index between soil layers and forest types in different seasons is analyzed by ANOVA combined with the multi-factor box plot of origin software.

### 2.7 Real-time fluorescence quantitative PCR

Real-time fluorescence quantitative PCR was performed to measure the 16S rRNA gene abundance of prokaryotic microorganisms in each soil. The primers 338F (5′-ACTCCTACGGGAGGCAGCA-3′) and 806R (5′-GGACTACHVGGGTWTCTAAT-3′) targeted 16S rRNA were used for real-time fluorescence quantitative PCR (Roche lightcycler96). The reaction included 10 μl 2 × AceQ Universal SYBR qPCR Master Mix (Vazyme, China), 0.4 μl former primer, 0.4 μl reverse primer, 2 μl 1‰ BSA (bovine serum albumin), 6.6 μl sterile water, and 0.6 μl DNA in 20 μl reaction system. The reaction was repeated three times for each sample. The cyclic conditions of this reaction were as follows: run at 95°C for 5 min; denatured at 95°C for 10 s, annealed at 54°C for 45 s, extended at 60°C for 45 s, a total of 40 cycles; extended at 72°C for 10 min. The reaction temperature increased from 65.0 to 95.0°C at a rate of 0.5°C s^−1^ during the analysis of the melting curve. The standard curve was constructed using a 10-fold serial dilution (10^2^-10^7^ gene copies μl^−1^) of newly extracted plasmids containing the corresponding gene fragments. The correlation coefficient *R*^2^ was higher than 99% for the standard curve.

### 2.8 Nucleotide sequence accession numbers

The raw sequence data of the 16S rRNA gene analyzed in this study was uploaded to the sequence read archive on the NCBI website under the accession number PRJNA992889.

## 3 Results

### 3.1 The effect of soil properties on prokaryotic diversity and abundance

**Figure 1:**
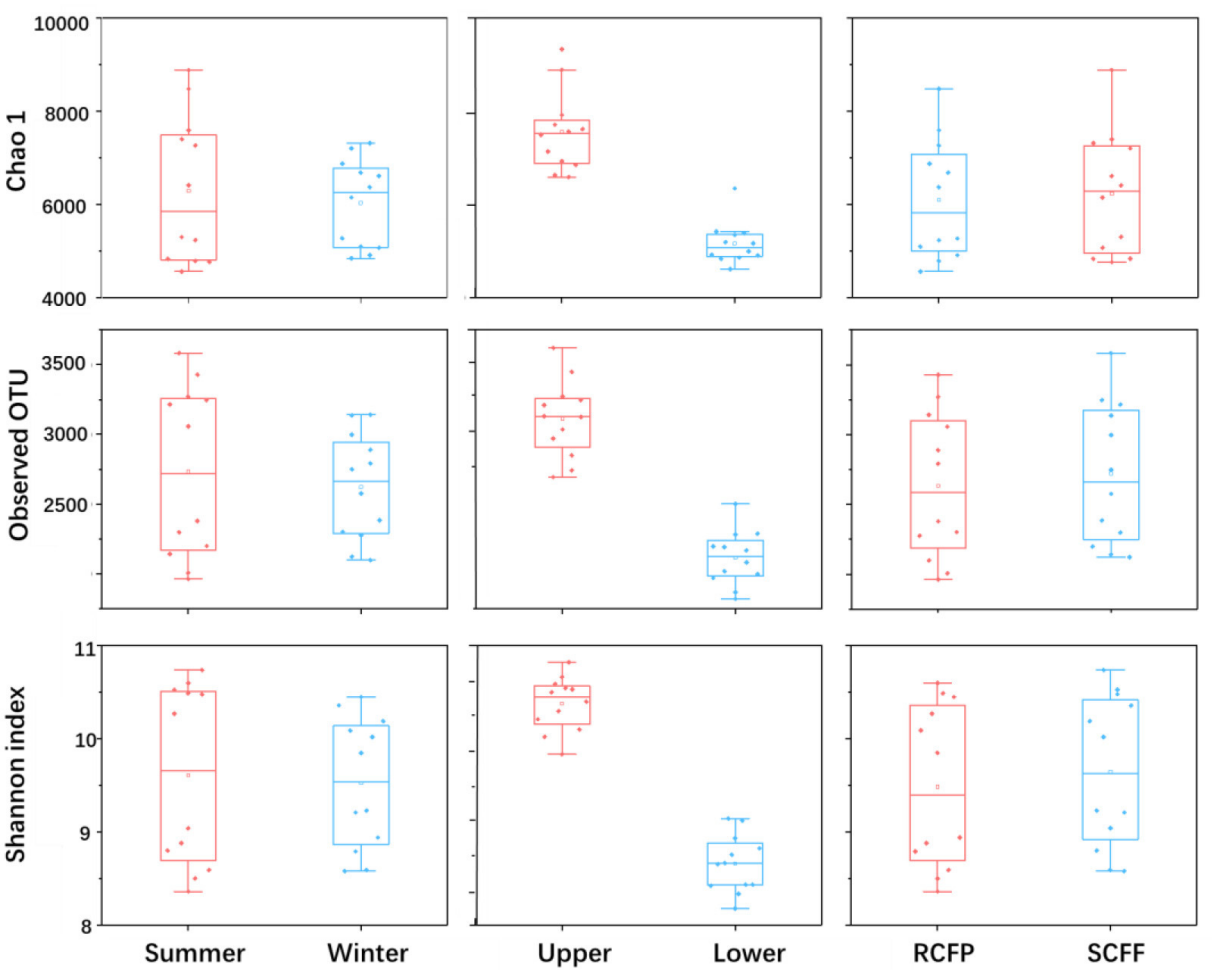
Effects of season, soil layer and forest type on alpha diversity of prokaryotic microorganisms in forest soils. The horizontal line in each box is the median value. Sample IDs: RCFP, reforested Chinese fir plantation; SCFF, secondary Chinese fir forest; Upper, upper layer; Lower, lower layer.

Chao1, OTU numbers, and Shannon index of the prokaryotic organisms are given in Table 1. Soil layer had a significant influence on α diversity of prokaryotic microorganisms (*p* < 0.01; Figure 1). The Shannon index was selected to analyze the relationship between prokaryotic diversity and soil factors. Pearson correlation coefficient analysis showed that soil organic matter, total N, hydrolyzable N and ${\text{NH}}_{4}^{+}-\text{N}$ were positively correlated with the Shannon index (*p* < 0.05; Table 2). Soil pH, total P, total K, available P, ${\text{NO}}_{3}^{-}-\text{N}$, ${\text{SO}}_{4}^{\text{2}-}$, and Al^3+^ were not significantly correlated with the Shannon index (*p* < 0.05). It was also evident that organic matter and ${\text{NO}}_{3}^{-}-\text{N}$ were positively correlated with prokaryotic abundance (*p* < 0.05; Table 2), while soil pH, total N, total P, total K, hydrolyzed N, available P, ${\text{NH}}_{4}^{+}-\text{N}$, ${\text{SO}}_{4}^{\text{2}-}$, and Al^3+^ were not significantly correlated with prokaryotic abundance (*p* < 0.05).

**Table 1:** Observation and estimation of prokaryotic community diversity based on 16S rRNA gene sequences (±) sign indicated standard error of mean of (*n* = 3).

Sample ID^*^	**Chao1**	**Observed OTU**	**Shannon index**
S_RCFP_u	7,780 ± 629	3,252 ± 187	10.45 ± 0.17
S_RCFP_l	4,857 ± 343	2,116 ± 229	8.58 ± 0.27
S_SCFF_u	7,566 ± 1248	3,348 ± 204	10.58 ± 0.14
S_SCFF_l	4,962 ± 292	2,212 ± 79	8.81 ± 0.23
W_RCFP_u	6,643 ± 256	2,941 ± 181	10.13 ± 0.30
W_RCFP_l	5,088 ± 180	2,225 ± 111	8.77 ± 0.18
W_SCFF_u	7,043 ± 380	2,961 ± 197	10.19 ± 0.17
W_SCFF_l	5,351 ± 700	2,361 ± 228	9.01 ± 0.37

^*^Sample ID: S, summer; W, winter; RCFP, reforested Chinese fir plantation; SCFF, secondary Chinese fir forest: u, upper layer; l, lower layer.

**Table 2:** Correlation coefficients between soil physicochemical parameters and prokaryotic diversity and abundance.

	**pH**	**Organic matter**	**Total N**	**Total P**	**Total K**	**Hydrolyzable N**	**Available P**	** ${\text{NO}}_{3}^{-}-\text{N}$ **	** ${\text{NH}}_{4}^{+}-\text{N}$ **	** ${\text{SO}}_{4}^{\text{2}-}$ **	**Al^3+^**	**Shannon index**
Organic matter	−0.053											
Total N	−0.023	**0.973** ^**^										
Total P	−0.608	0.424	0.416									
Total K	**0.717** ^*^	−0.021	−0.074	−0.075								
Hydrolyzable N	0.005	**0.907** ^**^	**0.912** ^**^	0.541	0.095							
Available P	0.100	−0.090	0.074	−0.140	−0.328	0.040						
${\text{NO}}_{3}^{-}-\text{N}$	0.301	0.646	0.540	−0.034	0.306	0.380	−0.309					
${\text{NH}}_{4}^{+}-\text{N}$	0.121	**0.938** ^**^	**0.905** ^**^	0.280	0.059	**0.910** ^**^	0.076	0.641				
${\text{SO}}_{4}^{\text{2}-}$	−0.424	−0.038	−0.066	0.499	−0.073	0.250	0.163	−0.511	0.056			
Al^3+^	0.603	0.461	0.437	−0.579	0.208	0.240	−0.060	0.670	0.506	−0.586		
Shannon index	0.076	**0.861** ^**^	**0.867** ^**^	0.506	0.161	**0.801** ^*^	−0.106	0.669	**0.780** ^*^	−0.249	0.293	
Prokaryotic abundance	−0.082	**0.802** ^*^	0.722^*^	0.270	−0.001	0.513	−0.399	**0.869** ^**^	0.654	0.502	−0.456	**0.796** ^*^

^*^Indicates significantly correlated at level *p* < 0.05.

^**^Indicates significantly correlated at level *p* < 0.01.

Bold values indicate statistical significance.

### 3.2 Relationship between soil parameters and the dominant families

The prokaryotic communities of all samples from the two Chinese fir forests on the family level mainly consisted of Koribacteraceae (15.38%), Thermogemmatisporaceae (5.36%), Chthoniobacteraceae (3.04%), Solibacteraceae (2.55%), and Rhodospirillaceae (2.06%), Acidobacteriaceae (1.95%), Sinobacteraceae (1.41%), Syntrophobacteraceae (1.12%), Pedosphaeraceae (1.10%), Hyphomicrobiaceae (0.63%), and other families (65.41%). Therefore, the relationship between these 10 most abundant families and soil factors were studied.

Pearson correlation coefficient analysis showed that the relative abundance of the 10 most abundant prokaryote families was significantly correlated with certain soil physicochemical parameters (Table 3). Organic matter was positively correlated with most families: Koribacteraceae, Hyphomicrobiaceae, Acidobacteriaceae, Sinobacteraceae, and Solibacteraceae (*p* < 0.05), while negatively correlated with Syntrophobacteraceae and Chthoniobacteraceae (*p* < 0.05). Total N was also a crucial factor, positively correlated with most families: Koribacteraceae, Hyphomicrobiaceae, Acidobacteriaceae, Sinobacteraceae, Solibacteraceae (*p* < 0.05), and negatively correlated with Chthoniobacteraceae (*p* < 0.05). ${\text{NO}}_{3}^{-}-\text{N}$ was also a key factor, with positive correlations with Koribacteraceae, Acidobacteriaceae, and Solibacteraceae (*p* < 0.05), while negative correlations with Syntrophobacteraceae and Chthoniobacteraceae (*p* < 0.05). Thermogemmatisporaceae was positively correlated with pH and negatively correlated with total P (*p* < 0.05). Hydrolyzable N was positively correlated with Hyphomicrobiaceae and Sinobacteraceae (*p* < 0.05). ${\text{NH}}_{4}^{+}-\text{N}$ was positively correlated with Koribacteraceae and Sinobacteraceae (*p* < 0.05), while negatively correlated with Chthoniobacteraceae. Al^3+^ had a negative correlation with Chthoniobacteraceae (*p* < 0.05). Total K, available P, and ${\text{SO}}_{4}^{\text{2}-}$ were not significantly correlated with any prokaryotic families. Rhodospirillaceae and Pedosphaeraceae were not significantly affected by any of the identified parameters.

**Table 3:** Correlation coefficients between relative abundance of families and soil parameters.

	**pH**	**Organic matter**	**Total N**	**Total P**	**Total K**	**Hydrolyzable N**	**Available P**	** ${\text{NO}}_{3}^{-}-\text{N}$ **	** ${\text{NH}}_{4}^{+}-\text{N}$ **	**Al^3+^**	** ${\text{SO}}_{4}^{\text{2}-}$ **
Rhodospirillaceae	0.543	0.067	0.267	−0.236	0.141	0.207	0.570	−0.196	0.098	0.291	−0.235
Koribacteraceae	−0.006	**0.862** ^**^	**0.797** ^*^	0.435	0.194	0.690	−0.461	**0.781** ^*^	**0.710** ^*^	0.413	−0.314
Thermogemmatisporaceae	**0.711** ^*^	−0.192	−0.090	**−0.820** ^*^	0.129	−0.185	0.519	−0.056	−0.027	0.605	−0.319
Syntrophobacteraceae	0.101	**−0.716** ^*^	−0.586	−0.530	−0.250	−0.600	0.526	**−0.765** ^*^	−0.647	−0.163	0.096
Chthoniobacteraceae	−0.522	**−0.747** ^*^	**−0.762** ^*^	0.077	−0.346	−0.596	−0.026	**−0.788** ^*^	**−0.738** ^*^	**−0.802** ^*^	0.488
Hyphomicrobiaceae	−0.108	**0.762** ^*^	**0.782** ^*^	0.688	0.113	**0.714** ^*^	−0.070	0.552	0.635	0.054	−0.145
Acidobacteriaceae	0.021	**0.792** ^*^	**0.750** ^*^	0.386	0.167	0.572	−0.301	**0.843** ^**^	0.648	0.410	−0.436
Sinobacteraceae	0.255	**0.757** ^*^	**0.788** ^*^	0.437	0.290	**0.808** ^*^	−0.041	0.531	**0.736** ^*^	0.251	−0.197
Solibacteraceae	0.396	**0.775** ^*^	**0.745** ^*^	0.101	0.434	0.603	−0.279	**0.841** ^**^	0.693	0.698	−0.466
Pedosphaeraceae	0.674	−0.346	−0.229	−0.571	0.309	−0.260	0.678	−0.114	−0.154	0.268	−0.143

^*^Indicates significantly correlated at level *p* < 0.05.

^**^Indicates significantly correlated at level *p* < 0.01.

Bold values indicate statistical significance.

Redundancy analysis showed correlations between soil parameters and the relative abundance of families (Supplementary Figure 1). Organic matter, total N, and ${\text{NO}}_{3}^{-}-\text{N}$ were shown to be closely correlated with the relative abundances of most families, which is congruent with the results of Pearson correlation coefficient analysis (Table 3).

### 3.3 Effect of reforestation, soil horizon, and season on prokaryotic family composition

**Figure 2:**
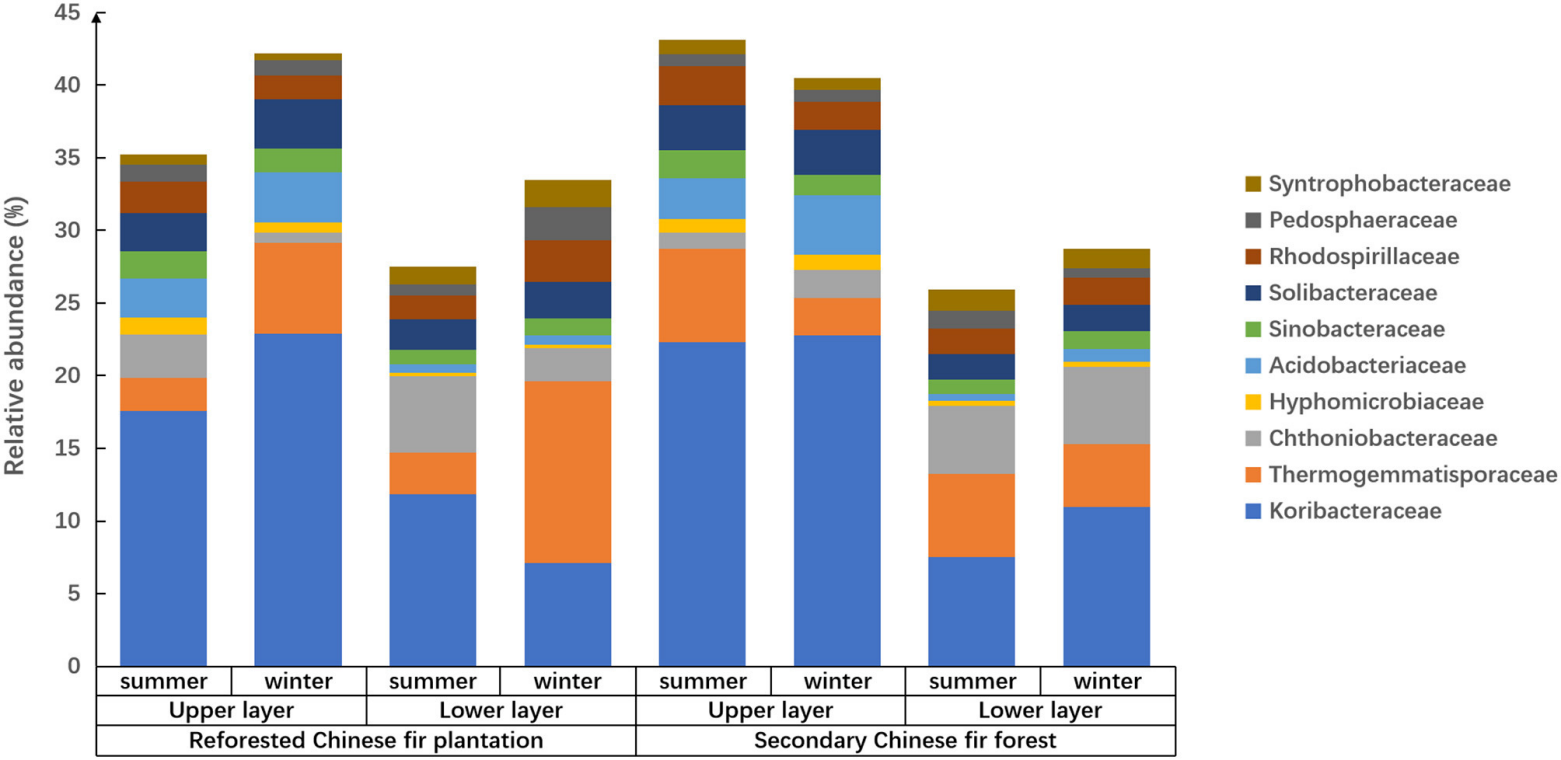
The relative abundance and composition of prokaryotes in soil of Chinese fir forests in subtropical Nanling National Nature Reserve at family level.

To explore the effect of reforestation, season, and soil horizon on prokaryote community structure, changes in relative abundances of the most abundant 10 families were explored and investigated when secondary Chinese fir forest was deforested and planted with Chinese fir seedlings. The results showed that the prokaryotic family composition of the soil samples showed different patterns between forest types, seasons, and soil layers (Figure 2).

Reforestation influenced the prokaryote composition because relative abundances of families were different between forest types, i.e., for the soils of the upper or lower horizons in summer, Koribacteraceae relative abundance was significantly higher in the secondary forest than the reforested plantation (*p* < 0.05; Figure 2 and Supplementary Table 2). In the upper layers of soil in winter, Thermogemmatisporaceae in the reforested Chinese fir plantation was higher than that in the secondary Chinese fir forest (*p* < 0.01). In the lower layers of soil in summer, Thermogemmatisporaceae in the secondary Chinese fir forest was higher than in the reforested Chinese fir plantation (*p* < 0.01). In the lower layers of soil in winter, Syntrophobacteraceae in the reforested Chinese fir plantation was higher than in the secondary Chinese fir forest (*p* < 0.01). Meanwhile, in summer in the lower horizons, Acidobacteriaceae in the reforested Chinese fir plantation was higher than in the secondary Chinese fir forest (*p* < 0.05). In the lower layers of soil in winter, Pedosphaeraceae in the reforested Chinese fir plantation was higher than in the secondary Chinese fir forest (*p* < 0.05).

**Figure 3:**
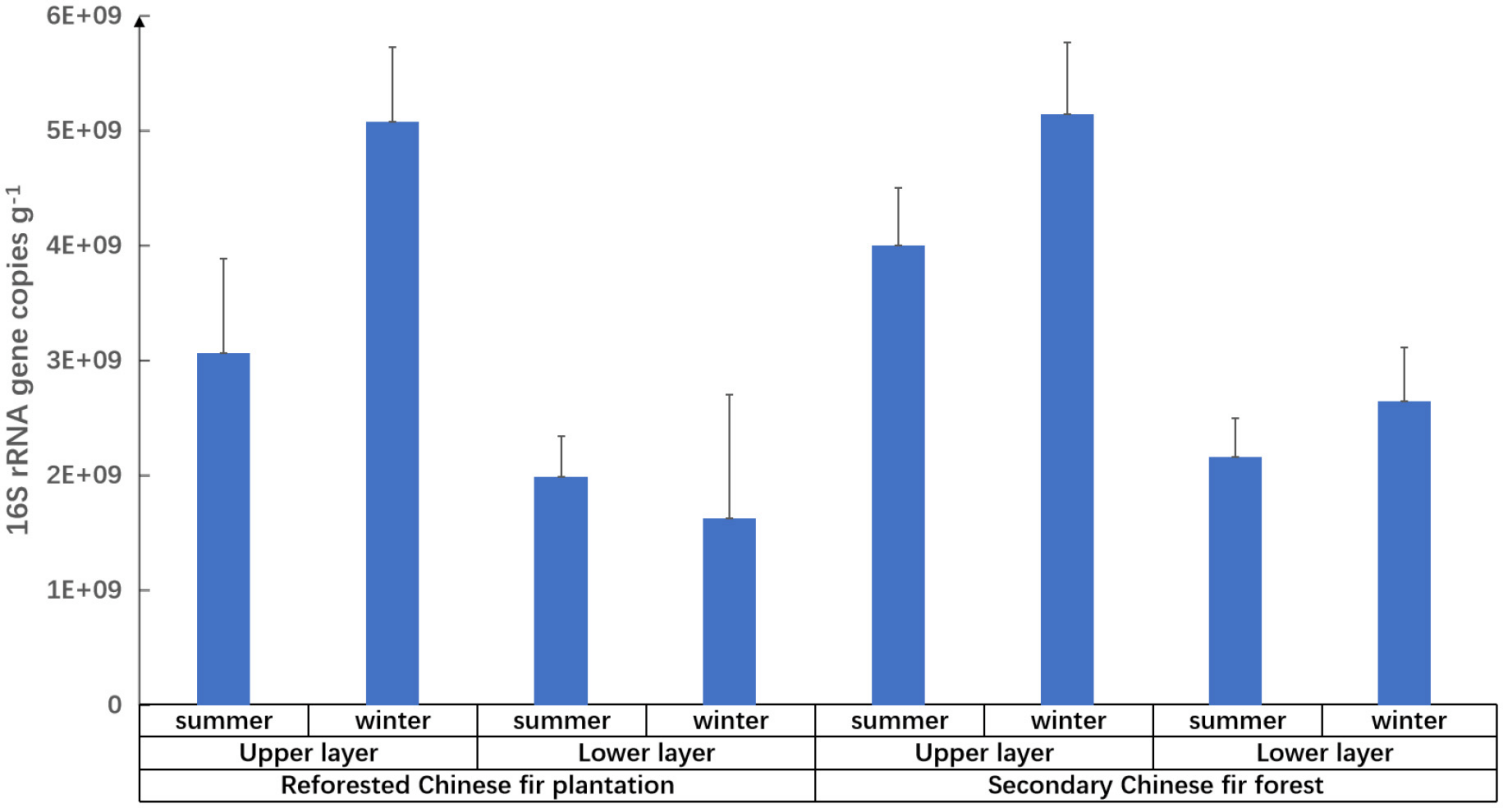
Relative abundance of prokaryotes in different types of Chinese fir forest soils in the Nanling National Nature Reserve.

Soil horizon significantly affected the family composition of the prokaryotic community because the relative abundances of different families in different soil layers varied among samples from the same site (Figure 2 and Table 4). The soil layer also affected the relative abundance of prokaryotes, which was twice as high in the upper soil layers as in the lower soil layers (Figure 3). The relative abundances of Koribacteraceae and Acidobacteriaceae in the upper layers of soil were higher than that in the lower layer at each site (*p* < 0.01). In summer and winter soils of the reforested Chinese fir plantation, and winter soils of the secondary Chinese fir forest, relative abundances of Syntrophobacteraceae were higher in the upper than in the lower layers (*p* < 0.01). Hyphomicrobiaceae, Solibacteraceae, Thermogemmatisporaceae, Chthoniobacteraceae, and Pedosphaeraceae also showed significant differences between upper and lower layers (*p* < 0.05; Figure 2 and Table 4).

**Table 4:** Dissimilarity of families between upper and lower soil layers (*n* = 3).

	**Rhodospirillaceae**	**Koribacteraceae**	**Thermogemmatisporaceae**	**Syntrophobacteraceae**	**Chthoniobacteraceae**	**Hyphomicrobiaceae**	**Acidobacteriaceae**	**Sinobacteraceae**	**Solibacteraceae**	**Pedosphaeraceae**
RCFP-sum	0.277	**0.003** ^**^	**0.029** ^*^	**0.002** ^**^	0.309	**0.008** ^**^	**0.002** ^**^	0.119	0.171	0.121
RCFP-win	0.206	**0.000** ^**^	0.173	**0.000** ^**^	0.083	0.087	**0.002** ^**^	**0.012** ^*^	0.184	**0.044** ^*^
SCFF-sum	0.098	**0.000** ^**^	0.710	0.134	**0.005** ^**^	**0.044** ^*^	**0.009** ^**^	0.059	**0.037** ^*^	0.753
SCFF-win	0.935	**0.005** ^**^	0.162	**0.009** ^**^	0.149	**0.012** ^*^	**0.009** ^**^	0.539	**0.016** ^*^	0.517

^*^Indicates significant difference between upper and lower soil layers at level *p* < 0.05.

^**^Indicates significant difference at level *p* < 0.01.

Sample ID: sum, summer; win, winter; RCFP, reforested Chinese fir plantation; SCFF, secondary Chinese fir forest.

Bold values indicate statistical significance.

The family composition and relative abundance of the prokaryotic community showed pronounced seasonality (Figure 2 and Supplementary Table 3). The relative abundance of prokaryotes in summer was at least 1.5 times higher than the relative abundance in winter for the same soil. In the same soil layers of each forest, prokaryotic community compositions were significantly different between seasons, especially in the reforested Chinese fir plantation. In the upper layers of the reforested Chinese fir plantation, Koribacteraceae in summer was significantly lower than in winter (*p* < 0.05); however, in the lower layer of reforested the Chinese fir plantation, Koribacteraceae in winter soil was significantly lower than in summer (*p* < 0.01). In the upper layer of the reforested Chinese fir plantation, Thermogemmatisporaceae in summer was significantly lower than in winter (*p* < 0.01). In addition, in the lower layers of the reforested Chinese fir plantation, Syntrophobacteraceae, and Pedosphaeraceae in summer were significantly lower than in winter (*p* < 0.05). In the upper layers of the reforested Chinese fir plantation, Chthoniobacteraceae in winter soil was significantly lower than in summer (*p* < 0.05).

### 3.4 Effect of reforestation, soil horizon, and season on the diversity and abundance of prokaryotic community

As can be seen from Supplementary Table 4, reforestation had no significant effect on the abundance or diversity of prokaryotes statistically. However, combined with Figure 2, reforestation changed the relative proportions of prokaryotic families, so reforestation affected community structure. Moreover, the pH of the secondary Chinese fir forest was significantly lower than that of reforested Chinese fir plantation (*p* < 0.05; Supplementary Table 4).

Prokaryotic diversity and abundance were significantly correlated with soil layers (Figure 3 and Table 5): prokaryotic biodiversity and abundance were higher in the uppers than the lower ones (*p* < 0.05), regardless of forest type or seasons.

**Table 5:** Dissimilarity of prokaryotic diversity, prokaryotic abundance, and soil parameters between upper and lower soil layers.

	**pH**	**Organic matter**	**Total N**	**Total P**	**Total K**	**Hydrolyzable N**	**Available P**	** ${\text{NO}}_{3}^{-}-\text{N}$ **	** ${\text{NH}}_{4}^{+}-\text{N}$ **	** ${\text{SO}}_{4}^{\text{2}-}$ **	**Al^3+^**	**Shannon index**	**Prokaryotic abundance**
RCFP-win	0.304	**0.006** ^**^	0.057	0.061	0.967	**0.015** ^*^	0.465	0.162	0.148	0.093	0.252	**0.003** ^**^	**0.009** ^*^
SCFF-win	0.596	**0.001** ^**^	**0.009** ^**^	**0.003** ^**^	0.592	0.124	0.956	**0.002** ^**^	0.054	0.487	0.432	**0.007** ^**^	**0.005** ^*^
RCFP-sum	0.445	**0.023** ^*^	**0.010** ^*^	0.290	0.833	**0.040** ^*^	0.420	**0.025** ^*^	0.073	**0.048** ^*^	0.587	**0.001** ^**^	0.108
SCFF-sum	0.176	**0.000** ^**^	**0.006** ^**^	0.445	0.511	**0.043** ^*^	0.693	**0.005** ^**^	0.268	**0.029** ^*^	**0.041** ^*^	**0.000** ^**^	**0.006** ^*^

^*^Indicates significant difference between upper and lower soil layers at level *p* < 0.05.

^**^Indicates significant difference at level *p* < 0.01.

Sample ID: sum, summer; win, winter; RCFP, reforested Chinese fir plantation; SCFF, secondary Chinese fir forest.

Bold values indicate statistical significance.

Season affected prokaryotic diversity at the family level (Supplementary Table 5). For the lower layers of the reforested Chinese fir plantation, prokaryotic diversity in summer was significantly higher than in winter (*p* < 0.05). The season had a certain degree of effect on prokaryotic abundance, with significant differences (*p* < 0.05) in the upper layers of the reforested Chinese fir plantation.

### 3.5 Effect of reforestation, soil horizon, and season on prokaryotic community structure by PCoA analysis

**Figure 4:**
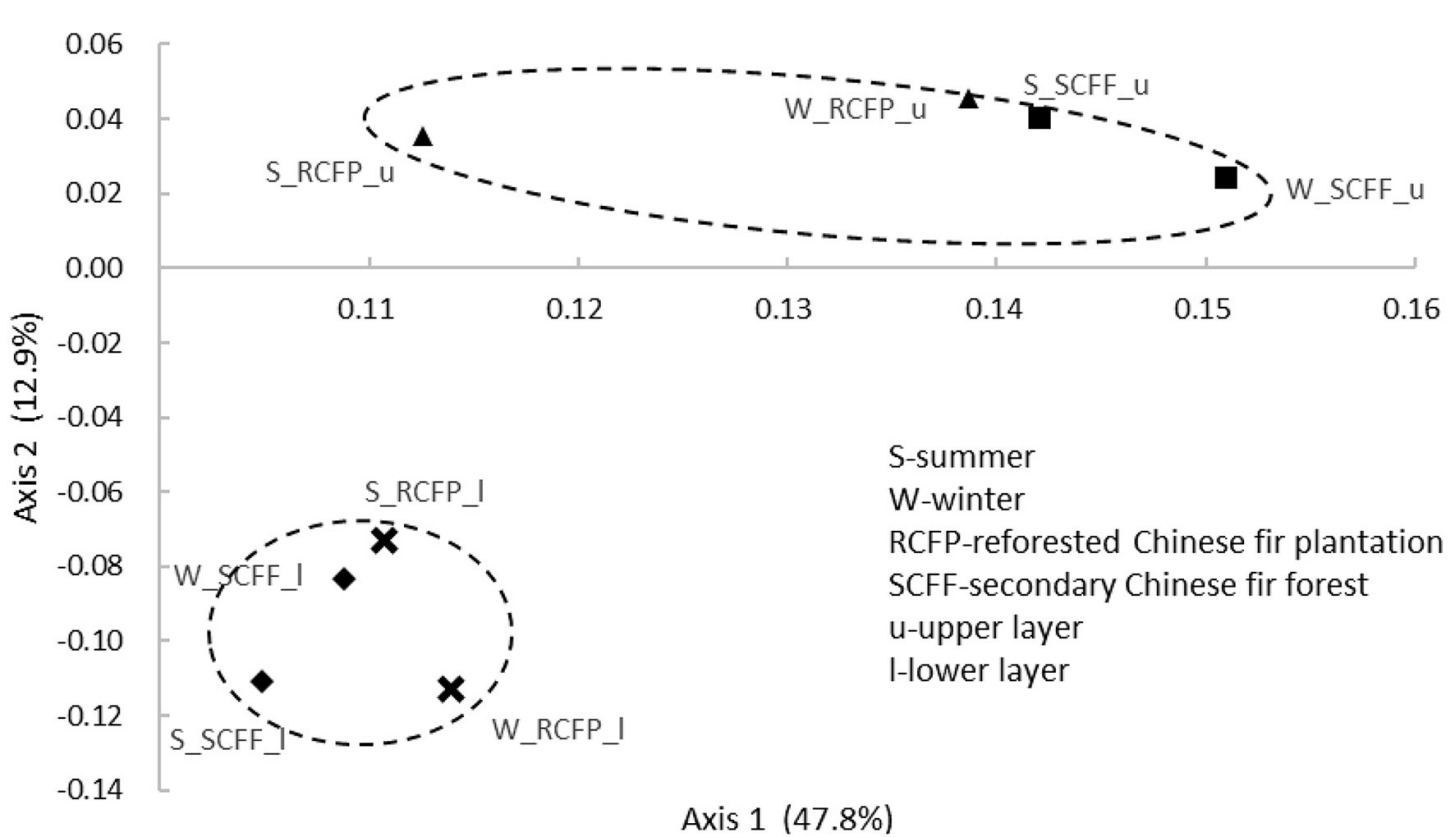
Principal coordinate analysis showing the effects of soil layer on prokaryotic community structure.

**Figure 5:**
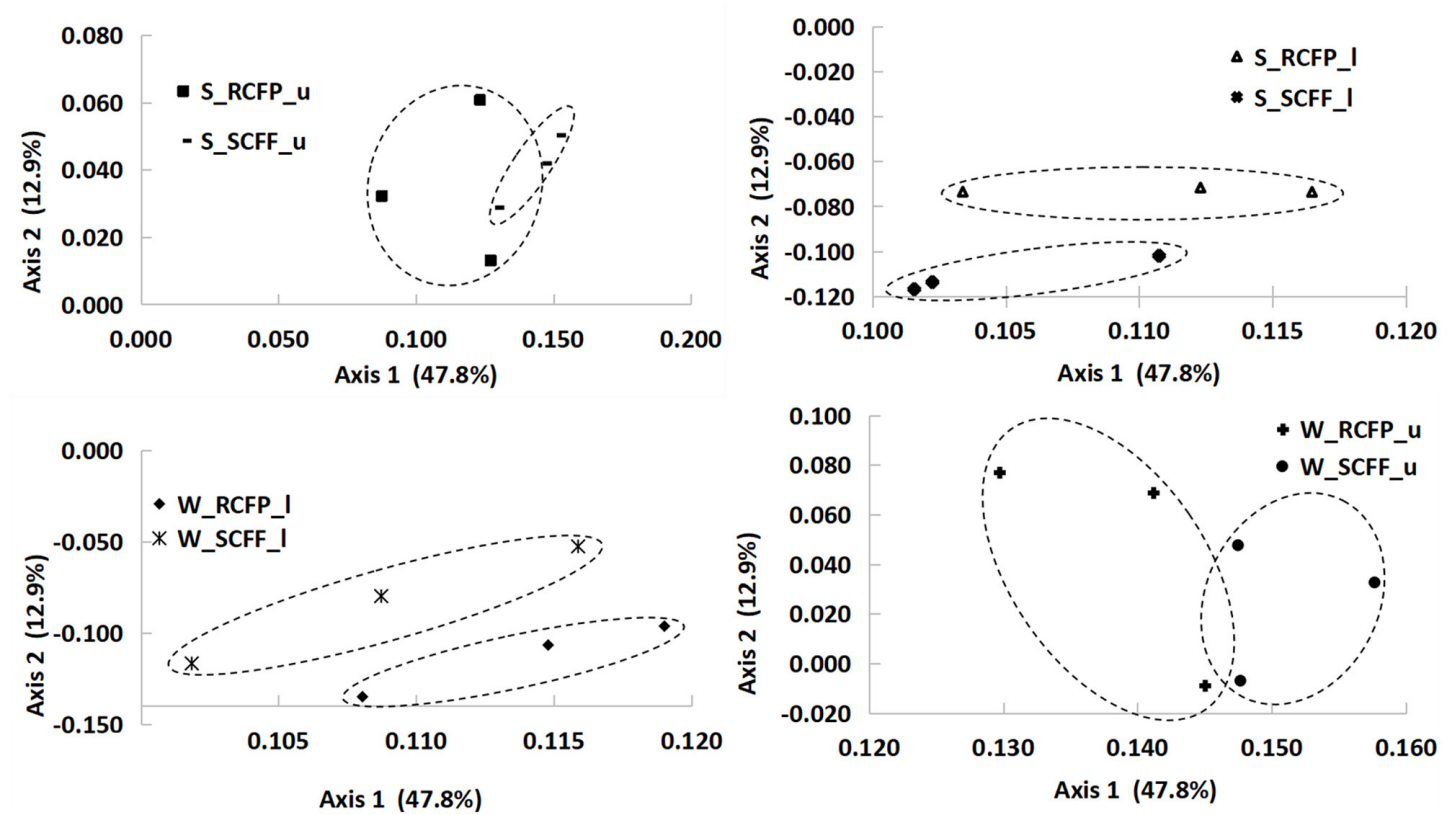
Principal coordinate analysis showing the effects of reforestation on prokaryotic community structure.

Principal coordinate analysis (PCoA) based on OTU data showed similarities in prokaryotic communities between soil types, soil layers, and seasons (Figures 4, 5 and Supplementary Figure 2). Reforestation had a strong influence on the community structure of prokaryotic organisms because the same soil layers in the same season were always different between the two forest types (Figure 5). Soil layer had a strong influence on the structure of the prokaryotic community because the upper and lower layers were separately clustered (Figure 4). Season had a certain degree of effect on community structure although samples from the same site in different seasons were relatively close (Supplementary Figure 2). In summary, PCoA analysis showed similar results as the above other methods.

## 4 Discussion

### 4.1 Reforestation changed prokaryotic community composition

The present study showed that the phylum Acidobacteria (including Koribacteraceae, Solibacteraceae, and Acidobacteriaceae) dominated in the soil of the two Chinese fir forests, accounting for 58.86% of the prokaryotic community (Supplementary Figure 3). This may be because Acidobacteria are oligotrophic (Fierer et al., 2007) and prefer low pH environments (Jones et al., 2009; Zhang et al., 2017), whereas in this experiment the two Chinese fir forest soil sample had a lower average pH (pH = 4.4), thus favoring the growth of the Acidobacteria phylum. This finding is in line with some studies, such as the study of Pankratov et al. (2008) that showed some members in the phylum Acidobacteria have an optimal growth pH of 3.5–4.5; *Ca*. Koribacter versatilis strain Ellin345 and *Ca*. Solibacter usitatus strain Ellin6076 in the Acidobacteria phylum grow best at pH 4.0 to 6.0 (Ward et al., 2009); three subgroups in Acidobacteria were found in forest samples in the pH range 3.5–5.0 (Kim et al., 2021).

The results showed that the community structure of prokaryotes changed when the secondary Chinese fir forest was clear-cut and reforested with Chinese fir seedlings. Importantly, the family level composition of soil prokaryotes changed greatly. This change might come from the difference in pH. The pH of the secondary Chinese fir forest was significantly lower than that of reforested Chinese fir plantation (*p* < 0.05). Therefore, Koribacteraceae (belonging to Acidobacteria) was significantly higher in the secondary Chinese fir forest soil than in the reforested Chinese fir plantation. Other studies (Eichorst et al., 2007; Hartman et al., 2008; Lauber et al., 2009) have also shown a significant increase in the abundance of Acidobacteria at lower pH (2–6). In the present study, the relative proportion of Proteobacteria (Rhodospirillaceae, Syntrophobacteraceae, Hyphomicrobiaceae, and Sinobacteraceae) increased with the decrease of anthropogenic disturbance: Proteobacteria accounted for a lower proportion in the prokaryotic community of reforested Chinese fir plantation than in secondary Chinese fir forest. Our results were congruent with previous studies (Jangid et al., 2013; Li et al., 2014), which showed that the numbers of proteobacteria increased over time after anthropogenic disturbance ceased.

The finding that forest conversion affects microbial communities composition is similar to the results of several previous studies, for example, in areas where native broadleaf forests were converted to plantation forests in Fujian Province, China (Guo et al., 2016a), the relative abundance of Gram-positive bacteria in the soil of the Chinese fir plantation forest was significantly lower than that in the soil of the native broad-leaved forest; in Zhejiang Province, China where broadleaf forests were converted to bamboo forests, anaerobic bacterial abundance increased significantly (Guo et al., 2016b); in Hunan Province, China after converting from a natural evergreen and deciduous broad-leaf forest to four different 5-year old monoculture plantations, the relative abundance of *Ca*. solidcharacter, *Acidibacter, Occallatibacter, Burkholderia*, and *Acidothermus* decreased, while the relative abundances of the genera HSB_OF53-F07 (Order: Ktedonobacterales) and FCPS473 (Family: Ktedonobacteraceae) increased (Liu et al., 2020); in Guangdong Province, China, the transition from the Masson pine to eucalyptus plantation forests resulted in a significant decrease in soil AOA abundance (Zhang et al., 2016); and in Sumatra, Indonesia, tropical rainforests have been transformed into rubber agroforestry composite forests, leading to a decrease in the abundance of Gram-negative bacteria (Krashevska et al., 2015).

The present study showed a change in microbial community composition; however, the abundance and diversity were not significantly different between the two forests. This may be due to an increase in some prokaryotic taxa, such as Rhodospirillaceae, Hyphomicrobiaceae, and Chthoniobacteraceae, and a decrease in others, such as Thermogemmatisporaceae and Solibacteraceae, which ultimately resulted in a statistically unaffected abundance and diversity of the prokaryotic community in these two Chinese fir forests. The above changes in the family level may be due to the following reasons. Soil organic matter, containing carbon, and nitrogen elements, is conducive to the growth of Alphaproteobacteria (e.g., Rhodospirillaceae and Hyphomicrobiaceae) and Gammaproteobacteria (e.g., Sinobacteraceae; Alonso-Saez et al., 2009), while high nitrogen concentrations are of no use to the growth of Acidobacteria (Koribacteraceae, Acidobacteriaceae, and Solibacteraceae; Wang et al., 2018). Another study demonstrated that soil organic matter produces α-D-lactose, which can facilitate the growth of Verrucomicrobia (including Chthoniobacteraceae and Pedosphaeraceae), whereas the abundance of Acidobacteria was significantly negatively correlated with total organic matter (Jeanbille et al., 2016).

### 4.2 Soil depth significantly affected prokaryotic diversity and prokaryotic community composition

In this study, soil depth had a significant influence on prokaryotic biodiversity and community composition: the prokaryotic biodiversity in the upper layers of soil was significantly higher than that in the lower layers, which was consistent with recent studies (Pang et al., 2009; Selvam et al., 2010). This phenomenon was possibly due to the changes in soil organic matter as the upper soils contained higher organic matter, which was mainly derived from the decomposition of litter (Llado et al., 2017). The higher organic matter could serve as a substrate for different types of heterotrophic microbes, resulting in higher prokaryotic diversity (Wagner and Byrd, 2004). Besides, Acidobacteria can decompose cellulose (Stursova et al., 2012), so there were significantly more Acidobacteria in the upper soil than lower due to high cellulose in the upper layers. In particular, Koribacteraceae in the upper layers of soil was significantly higher than the lower ones, which might be because the extractable Al^3+^ in the upper layers of soil was higher than the lower ones. A former study also found a higher abundance of Koribacteraceae in tilled soil of higher extractable Al^3+^ (Lewis et al., 2018). The mechanism of why Koribacteraceae is positively related to Al^3+^ is still unknown. Our finding is that soil layers had a significant effect on prokaryotic diversity and composition, which is similar to other studies. Wang et al. (2022) showed that prokaryotic diversity was greater in the upper than the lower layers of the forest soil and that the prokaryotic community composition was different. Ren et al. (2023) also found a difference in the structure of the microbial community between different soil layers.

### 4.3 Seasonality pattern of the prokaryotic community composition

This study shows that prokaryotic community composition presented obvious seasonality, which is consistent with previous studies. For example, seasons influence the composition of Acidobacteria phylum in rhizospheres (Conradie and Jacobs, 2020); microbial community composition in riverbed sediments is influenced by seasons (Danczak et al., 2016); seasons alter halophilic prokaryotic communities in ponds at the Sfax solar saltern (Boujelben et al., 2012); seasons change prokaryotic communities in marine-snow-associated and ambient-water (Vojvoda et al., 2014).

In this study, the abundances of Rhodospirillaceae, Koribacteraceae, Thermogemmatisporaceae, Syntrophobacteraceae, Acidobacteriaceae, Solibacteraceae, and Pedosphaeraceae were higher in winter than in summer, whereas the abundances of Chthoniobacteraceae, Hyphomicrobiaceae, and Sinobacteraceae were higher in summer than in winter. The underlying mechanism might be related to the changes in temperature between seasons. Temperature changes alter the composition of bacterial communities in the soil (Fu et al., 2023). Dedysh and Sinninghe-Damste (2018) have shown that most Acidobacteria (Koribacteraceae, Acidobacteriaceae, and Solibacteraceae) either are classified as mesophiles or psychrotolerants with slow metabolic activity, that is, low temperature does not inhibit them as other microorganisms. Moreover, Jin and Mullens (2014) show that air temperature is positively correlated with the upper soil temperature. Therefore, our study showed that prokaryotes in the upper soils presented higher seasonality than those in the lower ones. Specifically, Koribacteraceae in winter soil was significantly higher than that in summer.

## 5 Conclusions

The results showed that forest reformation changed the composition of the prokaryotic community in soil. Soil depth had an important influence on the diversity of prokaryotes and the composition of the prokaryotic community: the diversity of prokaryotes was significantly higher in the upper layers of the soil than in the lower ones (*p* < 0.05). Season affected the composition of the prokaryotic community. In addition, the results showed that multiple phyla of soil microorganisms were found in Chinese fir forests with the dominance of Koribacteraceae (15.38%) from the phylum Acidobacteria (58.86%). Soil organic matter, total N, hydrolyzable N, and ${\text{NH}}_{4}^{+}-\text{N}$ were significantly correlated with prokaryotic diversity (*p* < 0.05). Organic matter and ${\text{NO}}_{3}^{-}-\text{N}$ were positively (*p* < 0.05) correlated with prokaryotic abundance. Overall, this study indicated that forest conversion transformed the soil prokaryotic community, which might be due to the changed soil physicochemical parameters.

## Data availability statement

The datasets presented in this study can be found in online repositories. The names of the repository/repositories and accession number(s) can be found at: NCBI—PRJNA992889.

## Author contributions

X-YH: Writing—original draft, Data curation, Formal analysis. W-TQ: Writing—original draft, Methodology. J-DG: Writing—review & editing, Investigation. C-YL: Writing—review & editing. MH: Writing—review & editing. D-LD: Writing—review & editing. YZ: Writing—review & editing. Y-FW: Conceptualization, Funding acquisition, Project administration, Investigation, Methodology. Writing—review & editing. QL: Writing—review & editing.
