# Supplementary material for: Reforestation of Cunninghamia lanceolata changes the relative abundances of important prokaryotic families in soil
Source: Front Microbiol. 2024 Feb 13;15:1312286. doi: 10.3389/fmicb.2024.1312286 (PMC10896735; doi:10.3389/fmicb.2024.1312286)
Supplement: Supplementary file 1 [file Data_Sheet_1.docx]

**Supplementary Material**

**Table S1** Soil physicochemical characteristics in the Chinese fir forests (n = 3)

| Season | Forest type | Layer | pH | Organic matter | Total N | Total P | Total K | Hydrolyzable N | Available P | NO_3_^-^-N | NH_4_^+^-N | SO_4_^2-^ | Exchangeable Al |
| --- | --- | --- | --- | --- | --- | --- | --- | --- | --- | --- | --- | --- | --- |
|  |  |  |  | -------------------------------g kg^-1^----------------------------- | | | | ---------------------------------------mg kg^-1^ ----------------------------------------- | | | | | cmol kg^-1^(1/3 Al^3+^) |
| Summer | Reforested Chinese fir plantation | Upper | 4.380±0.125 | 64.833±7.087 | 2.534±0.207 | 0.409±0.064 | 12.800±0.500 | 323.333±55.103 | 4.297±2.099 | 9.988±1.626 | 46.674±11.965 | 23.600±1.803 | 6.947±0.731 |
|  |  | Lower | 4.317±0.035 | 45.367±6.214 | 1.685±0.244 | 0.353±0.046 | 12.733±0.115 | 219.667±22.898 | 3.103±0.940 | 4.546±2.135 | 28.397±5.332 | 34.800±6.651 | 7.207±0.223 |
|  | Secondary Chinese fir forest | Upper | 4.363±0.112 | 96.433±0.569 | 3.643±0.290 | 0.362±0.038 | 12.167±1.25 | 429.333±90.732 | 4.180±0.596 | 9.360±0.881 | 63.681±21.119 | 20.100±8.007 | 8.623±0.285 |
|  |  | Lower | 4.257±0.006 | 59.100±3.601 | 2.286±0.325 | 0.333±0.046 | 11.633±0.289 | 260.333±41.356 | 5.007±3.322 | 5.989±0.606 | 45.739±11.786 | 37.633±4.409 | 7.457±0.615 |
| Winter | Reforested Chinese fir plantation | Upper | 4.453±0.071 | 80.467±9.281 | 2.72±0.268 | 0.277±0.008 | 12.763±1.645 | 297.000±31.000 | 3.380±0.493 | 20.110±13.291 | 58.706±26.846 | 23.100±7.663 | 9.424±0.387 |
|  |  | Lower | 4.510±0.044 | 39.800±9.070 | 1.881±0.478 | 0.232±0.030 | 12.683±2.685 | 178.000±40.262 | 4.833±3.078 | 6.969±0.276 | 30.387±5.355 | 33.600±3.148 | 8.650±0.923 |
|  | Secondary Chinese fir forest | Upper | 4.257±0.095 | 75.833±7.081 | 2.863±0.303 | 0.378±0.022 | 12.017±0.578 | 258.333±70.231 | 3.817±1.065 | 13.560±1.847 | 42.109±9.214 | 33.200±10.573 | 8.014±1.063 |
|  |  | Lower | 4.307±0.117 | 40.900±3.208 | 1.728±0.274 | 0.295±0.005 | 11.777±0.419 | 163.000±47.791 | 3.757±1.399 | 5.541±0.492 | 25.611±5.216 | 27.800±6.149 | 7.461±0.272 |

**Table S2** Dissimilarity of top ten families between reforested Chinese fir plantation and secondary Chinese fir forest (n = 3)

| Sample ID^#^ | Rhodospirillaceae | Koribacteraceae | Thermogemmatisporaceae | Syntrophobacteraceae | Chthoniobacteraceae | Hyphomicrobiaceae | Acidobacteriaceae | Sinobacteraceae | Solibacteraceae | Pedosphaeraceae |
| --- | --- | --- | --- | --- | --- | --- | --- | --- | --- | --- |
| RCFP-SCFF(sum-upp) | 0.363 | **0.014^*^** | 0.065 | 0.199 | 0.067 | 0.538 | 0.884 | 0.981 | 0.274 | 0.096 |
| RCFP-SCFF(win-upp) | 0.372 | 0.921 | **0.007^**^** | 0.063 | 0.110 | 0.190 | 0.470 | 0.580 | 0.389 | 0.130 |
| RCFP-SCFF(sum-low) | 0.639 | **0.015^*^** | **0.004^**^** | 0.337 | 0.780 | 0.482 | **0.023^*^** | 0.974 | 0.336 | 0.108 |
| RCFP-SCFF(win-low) | 0.304 | 0.119 | 0.102 | **0.006^**^** | 0.190 | 0.341 | 0.494 | 0.814 | 0.305 | **0.025^*^** |

^*^ Indicates significant difference between reforested Chinese fir plantation and secondary Chinese fir forest at level *p* < 0.05

^**^ Indicates significant difference at level *p* < 0.01

^#^Sample ID: sum, summer; win, winter; RCFP, reforested Chinese fir plantation; SCFF, secondary Chinese fir forest; upp, upper layer; low, lower layer

**Table S3** Dissimilarity of the top ten families between summer and winter (n = 3)

| Sample ID^#^ | Rhodospirillaceae | Koribacteraceae | Thermogemmatisporaceae | Syntrophobacteraceae | Chthoniobacteraceae | Hyphomicrobiaceae | Acidobacteriaceae | Sinobacteraceae | Solibacteraceae | Pedosphaeraceae |
| --- | --- | --- | --- | --- | --- | --- | --- | --- | --- | --- |
| RCFP-upp | 0.327 | **0.011^*^** | **0.000^**^** | 0.122 | **0.038^*^** | 0.169 | 0.176 | 0.445 | 0.088 | 0.267 |
| RCFP-low | 0.193 | **0.005^**^** | 0.065 | **0.002^**^** | 0.190 | 0.727 | 0.053 | 0.615 | 0.536 | **0.031^*^** |
| SCFF-upp | 0.102 | 0.786 | 0.100 | 0.478 | 0.249 | 0.646 | 0.165 | 0.243 | 0.935 | 0.753 |
| SCFF-low | 0.803 | 0.168 | 0.175 | 0.691 | 0.757 | 1.000 | 0.239 | 0.567 | 0.854 | 0.065 |

^*^ Indicates significant difference between summer and winter at level *p* < 0.05.

^**^ Indicates significant difference at level *p* < 0.01.

^#^Sample ID: RCFP, reforested Chinese fir plantation; SCFF, secondary Chinese fir forest; upp, upper layer; low, lower layer

**Table S4** Dissimilarity of prokaryotic biodiversity and soil characters between reforested Chinese fir plantation and secondary Chinese fir forest (n = 3)

| Sample ID^#^ | pH | Organic matter | Total N | Total P | Total K | Hydrolyzable N | Available P | NO_3_^-^-N | NH_4_^+^-N | SO_4_^2-^ | Al^3+^ | Shannon index | Prokaryotic abundance |
| --- | --- | --- | --- | --- | --- | --- | --- | --- | --- | --- | --- | --- | --- |
| RCFP-SCFF(sum-upp) | **0.045^*^** | 0.530 | 0.573 | **0.002^**^** | 0.499 | 0.432 | 0.554 | 0.445 | 0.368 | 0.251 | 0.097 | 0.779 | 0.911 |
| RCFP-SCFF(sum-low) | 0.872 | **0.002^**^** | **0.006^**^** | 0.340 | 0.461 | 0.159 | 0.931 | 0.588 | 0.292 | 0.501 | **0.021^*^** | 0.359 | 0.166 |
| RCFP-SCFF(win-upp) | **0.048^*^** | 0.853 | 0.656 | **0.021^*^** | 0.594 | 0.699 | 0.611 | **0.012^*^** | 0.330 | 0.220 | 0.099 | 0.379 | 0.208 |
| RCFP-SCFF(win-low) | **0.043^*^** | **0.03^*^** | 0.062 | 0.621 | 0.004^**^ | 0.210 | 0.394 | 0.323 | 0.081 | 0.572 | 0.544 | 0.320 | 0.573 |

* Indicates significant difference reforested Chinese fir plantation and secondary Chinese fir forest at level *p* < 0.05

** Indicates significant difference at level *p* < 0.01

^#^Sample ID: sum, summer; win, winter; RCFP, reforested Chinese fir plantation; SCFF, secondary Chinese fir forest; upp, upper layer; low, lower layer

**Table S5** Dissimilarity in prokaryotic biodiversity and soil properties between summer and winter (n = 3)

| Sample ID^#^ | pH | Organic matter | Total N | Total P | Total K | Hydrolyzable N | Available P | NO_3_^-^-N | NH_4_^+^-N | SO_4_^2-^ | Al^3+^ | Shannon index | Prokaryotic abundance |
| --- | --- | --- | --- | --- | --- | --- | --- | --- | --- | --- | --- | --- | --- |
| RCFP-upp | 0.427 | 0.081 | 0.395 | **0.024^*^** | 0.972 | 0.511 | 0.502 | 0.261 | 0.517 | **0.007^**^** | **0.000^**^** | 0.180 | **0.029^*^** |
| RCFP-low | 0.277 | **0.007^**^** | **0.032^*^** | 0.570 | 0.860 | 0.061 | 0.633 | **0.024^*^** | 0.180 | 0.392 | **0.023^*^** | **0.036^*^** | 0.069 |
| SCFF-upp | **0.004^**^** | 0.430 | 0.562 | **0.018^*^** | 0.976 | 0.194 | 0.405 | 0.123 | 0.672 | 0.058 | **0.001^**^** | 0.356 | 0.609 |
| SCFF-low | 0.501 | **0.003^**^** | 0.085 | 0.224 | 0.651 | 0.056 | 0.580 | 0.376 | 0.054 | 0.992 | **0.000^**^** | 0.475 | 0.222 |

* Indicates significant difference between summer and winter at level *p* < 0.05

** Indicates significant difference at level *p* < 0.01

^#^Sample ID: RCFP, reforested Chinese fir plantation; SCFF, secondary Chinese fir forest; upp, upper layer; low, lower layer


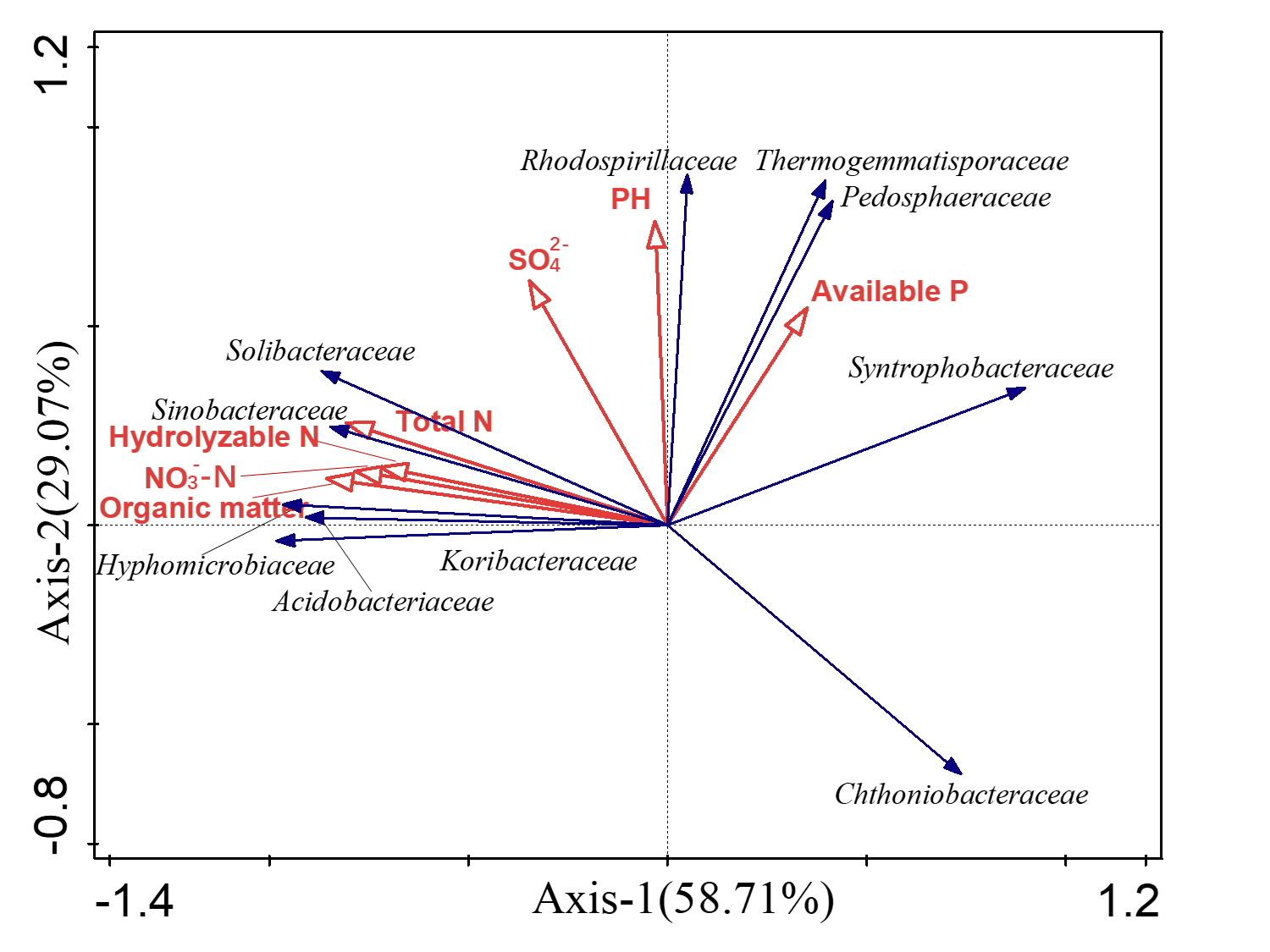


**Fig. S1** Redundancy analysis (RDA) showing the relationship between soil physicochemical parameters (red arrows) and prokaryotic families (blue arrows)


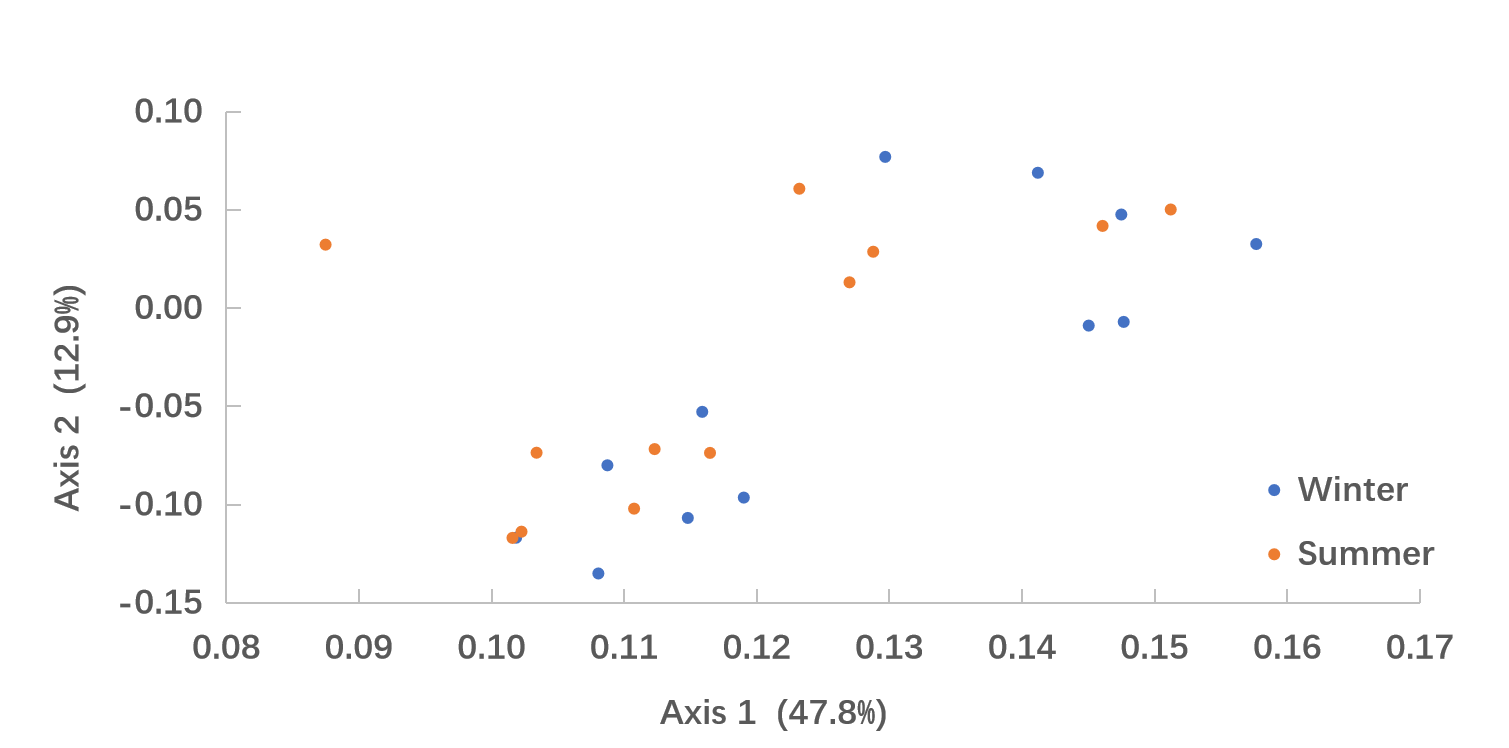


**Fig. S2** Principal coordinate analysis showing the effects of season on prokaryotic community structure


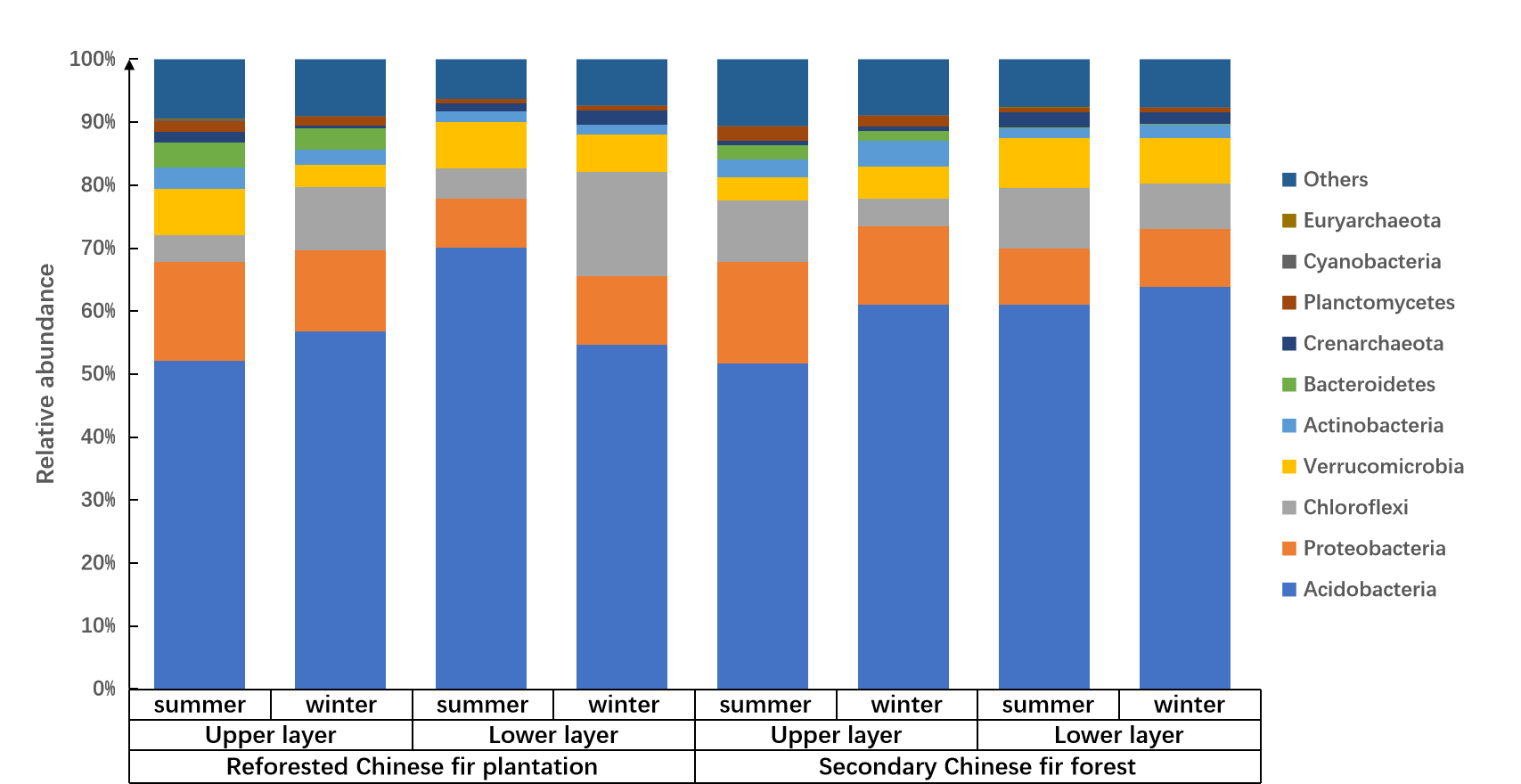


**Fig. S3** The phylum relative abundance in soils of Chinese fir forests in subtropical Nanling National Nature Reserve
